# Improved survival after treatments of patients with nonalcoholic fatty liver disease associated hepatocellular carcinoma

**DOI:** 10.1038/s41598-020-66507-7

**Published:** 2020-06-18

**Authors:** Jihane N. Benhammou, Elizabeth S. Aby, Gayaneh Shirvanian, Kohlett Manansala, Shehnaz K. Hussain, Myron J. Tong

**Affiliations:** 1 Pfleger Liver Institute, University of California Los Angeles, Los Angeles, CA USA; 2 Vatche and Tamar Manoukian Division of Digestive Diseases, David Geffen School of Medicine at UCLA, Los Angeles, California, USA; 3 Department of Epidemiology, Fielding School of Public Health, University of California, Los Angeles, CA USA; 4 Department of Medicine, Samuel Oschin Comprehensive Cancer Institute, Cedars-Sinai Medical Center, Los Angeles, CA USA; 5 Liver Center, Huntington Medical Research Institutes, Pasadena, CA USA

**Keywords:** Gastroenterology, Hepatology

## Abstract

Worldwide, nonalcoholic fatty liver disease (NAFLD) has reached epidemic proportions and in parallel, hepatocellular carcinoma (HCC) has become one of the fastest growing cancers. Despite the rise in these disease entities, detailed long-term outcomes of large NAFLD-associated HCC cohorts are lacking. In this report, we compared the overall and recurrence-free survival rates of NAFLD HCC cases to patients with HBV and HCV-associated HCC cases. Distinguishing features of NAFLD-associated HCC patients in the cirrhosis and non-cirrhosis setting were also identified. We conducted a retrospective study of 125 NAFLD, 170 HBV and 159 HCV HCC patients, utilizing clinical, pathological and radiographic data. Multivariate regression models were used to study the overall and recurrence-free survival. The overall survival rates were significantly higher in the NAFLD-HCC cases compared to HBV-HCC (HR = 0.35, 95% CI 0.15–0.80) and HCV-HCC (HR = 0.37, 95% CI 0.17–0.77) cases. The NAFLD-HCC patients had a trend for higher recurrence-free survival rates compared to HBV and HCV-HCC cases. Within the NAFLD group, 18% did not have cirrhosis or advanced fibrosis; Hispanic ethnicity (OR = 12.34, 95% CI 2.59–58.82) and high BMI (OR = 1.19, 95% CI 1.07–1.33) were significantly associated with having cirrhosis. NAFLD-HCC cases were less likely to exhibit elevated serum AFP (p < 0.0001). After treatments, NAFLD-related HCC patients had longer overall but not recurrence-free survival rates compared to patients with viral-associated HCC. Non-Hispanic ethnicity and normal BMI differentiated non-cirrhosis versus cirrhosis NAFLD HCC. Further studies are warranted to identify additional biomarkers to stratify NAFLD patients without cirrhosis who are at risk for HCC.

## Introduction

The metabolic syndrome, defined by the clustering of biochemical and clinical features, which includes type 2 diabetes (T2D), hypertension, dyslipidemia and obesity, has increased to epidemic proportions^1^. Non-alcoholic fatty liver disease (NAFLD), the liver manifestation of the metabolic syndrome, has increased in parallel and is now the most common cause of liver disease in the United States^2^. NAFLD can lead to nonalcoholic steatohepatitis (NASH), fibrosis, cirrhosis, and finally, hepatocellular carcinoma (HCC)^3^. NAFLD and its complications are predicted to continue to increase over the next decade, which is a reflection of the high prevalence of obesity/diabetes, progression of disease, and the aging patient population^4^. Therefore, NAFLD will have a growing burden on society, especially given the lack of optimal current therapies for NASH.

HCC burden has also shown an increase, doubling in incidence and mortality over the last decade, which has placed economic strains on health care^5^. Historically, chronic viral hepatitis etiologies were the main drivers of HCC; however, more recently, NAFLD and related metabolic factors have emerged as the most dominant risk factors with the highest population attributable fraction^6–8^. Although, the majority of NAFLD and NASH-related HCC cases occur in a cirrhosis background, non-cirrhosis HCC cases have been described to occur in up to 50% of cases^9,10^. Features of the metabolic syndrome - more specifically T2D - are highly associated with HCC development^11^. In a 26-year follow-up study from the Nurses’ Health Study and Health Professional Follow-up Study, the duration of T2D and the number of features of the metabolic syndrome were associated with HCC development in patients with and without cirrhosis^12^. Other large epidemiological HCC studies have also been instrumental in developing the understanding of clinical outcomes of the different causes of HCC^6^. However, the gold standard of detailed liver histology to diagnose NAFLD and NASH tend to lack from such large epidemiological reports^13^. Consequently, smaller studies with well-characterized phenotypic data have helped shed more light on the nuances of NAFLD HCC, although their small sample sizes and short follow up times have limited their generalizability^14,15^. As orthotopic liver transplantation (OLT) remains one of the only curative treatment for all etiologies of HCC^16^, understanding the natural history of HCC must be done in the context of regional variations of OLT^17,18^. Accordingly, there is an unmet need to study the implications of NAFLD and NASH in both cirrhotic and non-cirrhotic HCC with detailed pathologic data in different geographical regions of the transplantation allocation system.

Early HCC detection has been shown to improve survival^19–21^. Current society guidelines only recommend HCC screening in patients with cirrhosis or those with high risk features of chronic hepatitis B (HBV) infection^22^. This creates a clinical dilemma given the number of patients with NAFLD, the increase in HCC incidence and the potential for cancer in non-cirrhosis population not currently targeted for screening^23^. Thus, identifying clinical risk factors in NAFLD-associated HCC may provide valuable insight into identification and stratification of an “at-risk” patient population with NAFLD that may benefit from screening.

Accordingly, we aimed to study the overall and recurrence-free survival rates of NAFLD-associated HCC in cirrhosis and non-cirrhosis patients and compared their overall and recurrence-free survival outcomes to chronic viral etiologies of HCC in a large, diverse liver transplantation center in Los Angeles.

## Materials and Methods

The study was approved by the Institutional Review Board of the University of California, Los Angeles (IRB#17-000015). All methods were performed in accordance with the relevant guidelines and regulations. Informed consent was waived given the retrospective nature of our study, as determined by the IRB.

### Data source

This is a retrospective case-case comparison of NAFLD-HCC (including NASH) with chronic viral hepatitis (HBV and HCV) associated HCC cases^24^. Our data source for the NAFLD-HCC cases were evaluated between 1/1/2000 to 12/31/2016 and comprised of the UCLA Jonsson Comprehensive Care Center (JCCC) cancer registry as well as review of liver surgery, hepatology, and oncology clinic patient visits identified in the UCLA Electronic Medical Records (EMR). For further details on data source and case definitions, please see supplemental material.

### NAFLD-, HBV-, and HCV-HCC case definitions

All adult NAFLD HCC cases (men and women ≥ 18 years) who did not report excessive alcohol consumption as defined by the AASLD guidelines (>21 standard drinks on average per week for men and >14 standard drinks on average in women) were included. NAFLD HCC cases included all patients with a diagnosis of NAFLD, NASH (as determined by a hepatologist and/or review of the histological data), and cryptogenic cirrhosis, given that many patients with NAFLD and NASH had previously been misclassified as having cryptogenic cirrhosis^3,25^. Those patients with an additional diagnosis of HBV (HBV positive surface antigen), HCV (positive HCV RNA or history of HCV treatment with or without sustained virological response after treatment), primary biliary cholangitis, primary sclerosis cholangitis, alpha-1 anti-trypsin, Wilson disease and hemochromatosis were excluded from the NAFLD-HCC case group. Patients with mixed HCC and cholangiocarcinoma on pathology report were also excluded.

HBV and HCV related HCC cases were identified from a previously created data set of patients evaluated at the Liver Center in Pasadena, CA from 1984 to 2014^19^. Of the 333 HBV and HCV cases, four HBV and HCV co-infected patients were excluded from the HBV- and HCV-HCC cases. Therefore, 125, 170 and 159 patients were included in the NAFLD, HBV and HCV cohorts, respectively. Of note, in the NAFLD cohort, one patient had possible autoimmune hepatitis versus NASH, two patients had a positive HCV antibody (but without a positive RNA PCR or a history of HCV treatment, thus indicating a false positive or spontaneous clearance in the past without the development of chronic hepatitis as confirmed by the treating hepatologist), and one patient was homozygous for C282Y mutation with elevated ferritin (>1000), but did not show evidence of iron overload on pathology.

HCC cases were defined as anyone with evidence of Li-RADS-5 lesions on a contrast-enhanced study with CT or MRI or evidence of HCC on liver biopsy or on evaluation of the explanted liver (including autopsy in the event of death). Alpha-fetoprotein (AFP) was considered elevated if it was ≥ 10 ng/ml.

### Baseline laboratory and clinical data

All patients who met inclusion criteria had laboratory and clinical data and body mass index (BMI) evaluated within 6 months of the time of HCC diagnosis. If patients had laboratory data after 6 months from the time of HCC diagnosis, they were included in the final recurrence analyses without their laboratory data. Hypertension, dyslipidemia, T2D and glucose intolerance were defined by diagnosis (by ICD codes or from review of cardiology notes) or being on a medication associated with that diagnosis. For patients with a diagnosis of dyslipidemia, statin usage was recorded.

### HCC tumor characteristics

Tumor number and size were collected for all patients from contrast-enhanced cross sectional imaging (MRI and CT). Pathology data (from biopsy, explant, or resections) were reviewed when available. Studies done outside UCLA (interpreted locally or outside the institution) were included if local imaging data were not available. Abdominal ultrasound data were excluded. Tumors’ sizes were determined using those meeting Li-RADS-5 criteria^26^. Tumors measuring <2 cm on initial imaging study were included if they were confirmed as HCC on subsequent studies by imaging or pathology data. HCC cases were classified using the Milan criteria (single lesion 5 cm, maximum of three lesions with none>3 cm) and by the University of California at San Francisco (UCSF) criteria (single lesion 6.5 cm, maximum of three lesions with none>4.5 cm, or a total tumor burden of 8 cm). Metastasis was determined based on abdominal CT or MRIs as well as CT chest and bone scans.

### HCC treatment

HCC treatments were recorded for all patients, including when multiple therapies when conducted. Since many patients had several therapies, we defined most definitive to least definitive treatments as follows: OLT, hepatic resection (hepatectomy, segmentectomy, lobectomy), RFA (radiofrequency ablation), percutaneous ethanol injection (PEI), trans-arterial TACE/Y-90, systemic therapy, and supportive care (including hospice).

### Outcomes

We identified outcomes that occurred during the time period starting from the most definitive treatment through recurrence-free survival (composite event of death or recurrence) or overall survival (without recurrence). This was conducted for all treatment groups with the exception of the “Supportive” group (since they did not receive any therapy). We defined recurrent cases using the modified Response Evaluation Criteria in Solid Tumors (mRECIST) criteria^27^ for those treated with trans-arterial chemoembolization (TACE) or Y-90. RESICT criteria^28^ were used for those treated with other modalities of locoregional therapy such as RFA. We excluded any lesions that did not meet Li-RADS-5 criteria or if a study was done outside of UCLA without contrast agent. HCC screening/surveillance was defined as bi-annual abdominal ultrasounds or other imaging modalities (contrast enhanced CT or MRI) conducted for HCC surveillance^29^.

### Statistical analysis

The p values for between group comparisons of continuous variables that did not follow the normal distribution were computed using the non-parametric Kruskal-Wallis method. The p values for comparing continuous variables such as age that followed the normal distribution were computing using a one way analysis of variance model. The p values for comparing binary data across groups were computed using Fisher’s exact test.

A Cox proportional hazard model was used to compare recurrence free survival and patient survival curves adjusted for covariates. The Hazard (event rate) ratio (HR) and its 95% confidence bounds under this model is reported. Linearity between the log hazard rate and age, the only continuous covariate, was assessed using restricted cubic splines (RCS). To assess the effect of time on OLT, separate Cox proportional hazard models were used before and after 1/1/2000.

## Results

### Demographic and clinical characteristics of NAFLD, HBV, and HCV cases

The mean age of the NAFLD-associated HCC cases was 64.8 years with a mean BMI of 30.5 kg/m^2^ (±8.1 kg/m^2^). The majority had hypertension (n = 85, 68%) and dyslipidemia (n = 43, 34%). Sixty nine percent (n = 86) had T2D (n = 82) or glucose intolerance (n = 4). The majority had T2D or glucose intolerance for ≥10 years (n = 33), with 16 patients having had the disease for 2–10 years and only 2 for 0–2 years. Of the patients with T2D, 33% (n = 28) were on insulin therapy. The median A1c was 6.1 (IQR 5.4–6.95); however, the majority were on therapy by the time of A1c analysis. The majority of patients self-identified as Hispanic (n = 52, 42%).

Demographics of all three groups are presented in Table 1. Unlike HBV and HCV cases who had mostly men (n = 135, 79%, and n = 97, 61%, respectively), NAFLD cases were equally distributed between men (n = 59, 47%) and women (n = 66, 53%). NAFLD cirrhosis patients were more likely to have decompensated liver disease with 44% of the cohort with a Child- Pugh score of B and C (n = 45), when compared to HBV who only comprised 29% (n = 50) and HCV 23% (n = 36) of the cohorts. This is consistent with more patients in the NAFLD group having hepatic encephalopathy compared to HBV and HCV (25% versus 11% and 6%, respectively; p < 0.0001) and ascites/volume overload (36% versus 2% and 15%, respectively; p < 0.0001). In the hepatitis cohort, 33 patients were on therapy for chronic HBV and 26 patients had previously been treated for chronic HCV.

**Table 1:** Demographics and clinical characteristics of NAFLD, HBV and HCV cases.

	NAFLD	HBV	HCV	*P* value
Males n, %	59 (47)	135 (79)	97 (61)	<0.0010
Mean age at HCC dx ± SD	64.8 ± 8.5	57.7 ± 12.7	65.9 ± 10.3	<0.0001
Hispanic ethnicity, n (%)	52 (42)	2 (1)	29 (18)	<0.0010
T2D and glucose intolerance, n (%)^*^	86 (69)	21 (13)	28 (18)	<0.0001
**Decompensation, n (%)**				
*HE*	31 (25)	19 (11)	10 (6)	<0.0001
*Ascites/volume overload*	45 (36)	3 (2)	25 (15)	<0.0001
Child-Pugh Score*				
*A*	44 (43)^**^	120 (71)	121 (77)	<0.0010
*B*	35 (34)	41 (24)	30 (19)	
*C*	10 (10)	9 (5)	6 (3.7)	
*Missing data*	11	0	2	
Median INR (IQR) ^*^	1.2 (11–1.3)	1.1 (1–1.2)	1.1 (1.1–1.3)	0.0001
Median AST (IQR) ^*^	45 (33–60)	65 (35–113)	84 (49–128)	<0.0001
Median ALT (IQR) ^*^	32 (21–45)	54 (32–84)	64 (38–114)	<0.0001
Median bilirubin (IQR) ^*^	1.2 (0.7–2.3)	0.9 (0.7–1.6)	1.1 (0.8–1.6)	0.0246
Screened for HCC, n (%)	59 (47)	79 (47)	95 (58)	0.1053
Family history HCC, n (%)	11 (9)	42 (25)	8 (5)	<0.0001

*at the time of HCC diagnosis;** excludes patients without cirrhosis (see Table 3). T2D = type 2 diabetes; HE = hepatic encephalopathy; INR = International National Ratios; AST = aspartate aminotransferase; ALT = alanine aminotransferase; HCC = hepatocellular carcinoma; IQR = interquartile range.

NAFLD cases also were more likely to have T2D or glucose intolerance (69%, p < 0.0001). The HCC screening rate between all three groups was not significantly different (p = 0.1053). Patients with HBV were more likely to have a family history of HCC (25%) when compared to NAFLD (9%) or HCV (5%). Patients with NAFLD were most likely to have lower AST and ALT levels (p < 0.0001) but higher total bilirubin levels (p = 0.0246).

### Tumor characteristics of NAFLD, HBV and HCV cases

NAFLD and HCV HCC cases were more likely to be within Milan and UCSF criteria for liver transplantation than the HBV group (Table 2). This was further confirmed when assessing the median size of the first tumor which was similar in the NAFLD and HCV groups (2.9 and 3 cm, respectively) but markedly larger in the HBV group at 4 cm (p = 0.0003) (Table 2). We found that NAFLD HCC patients were less likely to have a positive AFP (AFP ≥ 10) when compared to the other two groups (p < 0.0001) (Table 2).

**Table 2:** Presenting tumor characteristics between NAFLD, HBV and HCV.

	NAFLD	HBV	HCV	*P* value
Within Milan (%)	85 (68)	79 (46)	109 (69)	<0.0001
Within UCSF (%)	100 (80)	93 (55)	128 (81)	<0.0001
Median first tumor size (cm) (IQR)	2.9 (2–4.5)	4 (2.4–7.6)	3 (2.1–4.6)	0.0003
Median tumor numbers (IQR)	1 (1–2)	1 (1–2)	1 (1–2)	0.232
AFP-producers	35 (35)	111 (66)	124 (76)	<0.0001
Most definitive therapy^*^, n (%):				
*OLT*	50 (40)	19 (11)	30 (19)	<0.001
*Resection*	14 (11)	37 (22)	14 (9)	
*RFA*	26 (21)	20 (12)	24 (15)	
*TACE/Y-90*	13 (10)	27 (16)	26 (15)	
*PEI*	0 (0)	2 (1)	6 (4)	
*Chemotherapy*	5 (4)	14 (8)	4 (3)	
*Supportive*	17 (14)	51 (30)	55 (35)	

IQR = interquartile range; AFP = alpha-fetoprotein; RFA = radiofrequency ablation; TACE = trans-arterial chemoembolization; OLT = orthotopic liver transplantation.

*Total group not sub-divided by OLT before or after 2000.

### Clinical and tumor characteristics of cirrhosis versus non-cirrhosis HCC cases

To characterize which clinical features were more likely to predict cirrhosis and advanced fibrosis in the NAFLD HCC cohort, we classified the group into “cirrhosis/advanced fibrosis” or “non-cirrhosis”. Cirrhosis/advanced fibrosis included anyone with clinical evidence of cirrhosis (platelets < 150 K)^30^, evidence of portal hypertension or as diagnosed by a hepatologist) or pathology review (all F3, F-4 and F4 on trichrome stain by METAVIR scoring system)^31^. The non-cirrhosis group was defined as anyone who had F0, F1-2, F2-3 disease on the pathology review or as determined by a hepatology clinic visit. A total of 86.5% of our cohort had pathology available for review. Ten patients (8%) had no evidence of clinical cirrhosis; 8 (6%) had F0; 2 (2%) had F0-1; 3 (2%) had F1-2; one patient had F2-3 (1%); 6 (5%) had F3; 6 (5%) had F3-4 and 90 (71%) had clinical evidence of cirrhosis or F4 disease on liver biopsy.

Based on these definitions, 102 (82%) had cirrhosis/advanced fibrosis and 23 (18%) had non-cirrhosis liver disease. The demographics comparing the two groups are presented in Table 3. In an unadjusted bivariate analysis comparing clinical factors associated with cirrhosis, we identified that being of non-Hispanic ethnicity (OR = 0.07, p = 0.0001), having dyslipidemia (OR = 0.321, p = 0.0268) and a lower BMI (p = 0.00065) were associated with non-cirrhosis in the NAFLD HCC group. Sixteen percent of patients (n = 16) in the cirrhosis/advanced fibrosis group were on statin therapy compared to 25% in the non-cirrhosis group (n = 5). HCC screening was higher in cirrhosis/advanced fibrosis group (p < 0.0001). Only one patient in the non-cirrhosis group was screened for HCC as 5 of his siblings had developed NAFLD-associated HCC.

**Table 3:** Clinical and tumor characteristics between the cirrhosis/advanced fibrosis versus non-cirrhosis group within the NAFLD cohort.

	Cirrhosis/advanced fibrosis (n = 102)	Non-cirrhosis (n = 23)	*P* value
Males n, %	45 (44)	14 (61)	0.170
Mean age at HCC dx ± SD	64.2 ± 7.4	67.1 ± 12	0.0334
Hispanic ethnicity	50 (49)	2 (9)	0.0001
Median BMI (IQR)	31.7 (28–34)	25.5 (22–30)	0.0002
T2D/GI, n (%)	72 (71)	14 (61)	0.458
Median A1c (IQR)	5.9 (5.4–6.9)	6.1 (5.4–6.5)	0.909
Hypertension, n (%)	67 (67)	17 (74)	0.470
Dyslipidemia, n (%)	30 (29)	13 (57)	0.0152
Statin use, n (%)	16 (16)	5 (25)	0.5383
**AFP-producers, n (%)**			
*Yes*	29 (29)	6 (26)	>0.999
*No*	63 (62)	14 (58)	
*Missing or no AFP*	8 (8)	3 (13)	
Screened, n (%)	58 (57)	1 (4)	<0.0001
FHx of HCC, n (%)	7 (7)	4 (17)	0.112
FHx LD, n (%)	28 (27)	4 (17)	0.423

SD = standard deviation; IQR = interquartile range; GI = glucose intolerance; T2D = type 2 diabetes; FHx=family history; AFP = alpha-fetoprotein; LD = liver disease.

In an adjusted multivariable analysis, patients who identified themselves as Hispanic (adj. OR = 12.34, 95% CI 2.59–58.82, p = 0.002) and who had a higher BMI (adj. OR = 1.19, 95% CI 1.07–1.33, p = 0.002) were more likely to have cirrhosis. T2D and glucose intolerance diagnoses showed an increased trend towards the cirrhosis group; however, the differences were not statistically significant (adj. OR = 1.46, 95% CI 0.46–4.61, p = 0.52) (Table 4) Similarly, age (adj. OR = 0.97, 95% CI 0.86–1.1, p = 0.47) and gender (adj. OR = 3.94, 95% CI 0.001–13261, p = 0.74) were not predictive of cirrhosis/advanced fibrosis, while dyslipidemia demonstrated a trend towards decreased cirrhosis (adj. OR = 0.54, 95% CI 0.164–1.76, p = 0.31).

**Table 4:** Odds ratios of clinical predictors of cirrhosis outcome in the NAFLD-associated HCC cohort.

	Unadjusted	*P* value	Adjusted^♯^	*P* value
Sex	1.97	0.170	—	—
Age*	—	0.286	—	—
BMI*	—	<0.001	1.162	0.010
Not Hispanic ethnicity	0.075	<0.001	0.091	0.003
T2D	1.59	0.478	1.464	0.515
Dyslipidemia	0.321	0.027	0.538	0.306

*Age and BMI analysis conducted in quartiles with student *t*-test for statistical testing. BMI = body mass index; T2D = type 2 diabetes.

^#^Model adjusted for ethnicity, dyslipidemia, T2D and BMI (per unit).

### Survival outcomes are not different between the NAFLD-, HBV- and HCV-HCC case groups

**Figure 1:**
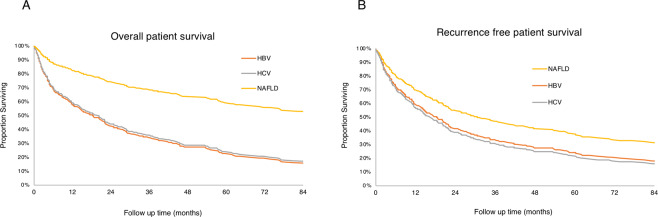
Overal and recurrence free survival of NAFLD, HBV and HCV cases. A. Overall survival of the three groups; B. Recurrence free survival for all three groups. The figure is adapted from Benhammou *et al*. from Bedside to Bench-side: the Clinical, Epidemiological and Molecular Basis for Nonalcoholic Steatohepatitis and Hepatocellular Carcinoma. *UCLA*^24^.

At a median follow up of 17 months, we found that NAFLD-HCC patients had improved overall survivals compared to the HCV patients (adj. HR = 0.37, 95% CI 0.17–0.77, p = 0.003) and HBV patients (adj. HR = 0.35, 95% CI 0.15–0.80, p = 0.013) after adjusting for age, gender, ethnicity, most definitive treatment and Child-Pugh score (Fig. 1, Table 5). As expected, the type of definitive therapy influenced the survival rates of the groups, with orthotopic liver transplantation (OLT) giving the most decrease in death or HCC recurrence rates (adj. HR = 0.08 and adj. HR = 0.09, respectively). In the adjusted recurrence-free models, NAFLD-HCC patients had a trend towards improved outcomes compared to HCV and HBV (adj. HR = 0.64 and adj. HR = 0.69, respectively). No differences were seen in the recurrence-free survival rates between the HCV and HBV groups (adj. HR = 1.08, 95% CI 0.78–1.49, p = 0.650).

**Table 5:** Cox multivariable analysis of patients and treatment variables associated with overall survival and recurrence free survival (n = 454).

Variable	HR	95% CI	*P* value
***Overall Survival***			
Male gender	0.16	0.88–1.54	0.2891
Age (per year)	0.99	0.98–1.01	0.0741
**Etiologies:**			
*HCV vs HBV*	0.96	0.68–1.34	0.8118
*NAFLD vs HBV*	0.35	0.15–0.80	0.0134
*NAFLD vs HCV*	0.37	0.17–0.77	0.0034
**Race/Ethnicity**			
*African American*	Ref	—	—
*White*	0.66	0.23–1.89	0.4379
*Asian*	0.74	0.26–2.05	0.5568
*Hispanic*	0.9	0.30–2.68	0.8478
*Not Hispanic*	1.52	0.39–5.86	0.5451
**Most definitive treatment:**			
*Chemotherapy*	Ref	—	—
*OLT (after year 2000)*	0.08	0.04–0.17	<0.0001
*OLT (before year 2000)*	0.11	0.04–0.27	<0.0001
*PEI*	0.36	0.14–0.92	0.0338
*Resection*	0.15	0.08–0.29	<0.0001
*RFA*	0.16	0.08–0.30	<0.0001
*TACE*	0.45	0.26–0.79	0.0055
*Supportive care*	0.83	0.49–1.42	0.5
***Recurrence Free Survival***			
Male gender	1.12	0.90–1.51	0.245
Age (per year)	0.99	0.98–1.01	0.0626
Etiologies:			
*HCV vs HBV*	1.08	0.78–1.49	0.6504
*NAFLD vs HBV*	0.69	0.39–1.39	0.3002
*NAFLD vs HCV*	0.64	0.34–1.20	0.163
**Ethnicity:**			
*African American*	Ref	—	—
*White*	0.76	0.27–2.18	0.6104
*Asian*	0.91	0.33–2.53	8608
*Hispanic*	0.92	0.31–2.71	0.8734
*Not Hispanic*	0.84	0.24–2.93	0.7824
**Most definitive treatment:**			
*Chemotherapy*	Ref	—	—
*OLT (after year 2000)*	0.09	0.05–0.17	<0.0001
*OLT (before year 2000)*	0.11	0.04–0.28	<0.0001
*PEI*	0.38	0.15–0.98	0.0461
*Resection*	0.24	0.14–0.44	<0.0001
*RFA*	0.28	0.16–0.51	<0.0001
*TACE*	0.54	0.31–0.93	0.0276
*Supportive Care*	0.75	0.43–1.26	0.2749

Harrell’s C-statistic =0.780 for the overall survival and 0.737 for the recurrence-free survival.

Given that many patients had OLT as a most definitive treatment and the significant improved survival rates with OLT, we further adjusted the model for the time of surgery to control for improvements in surgical and medical techniques. We stratified our data by assessing the survival rates before and after the year 2000 and found that OLT remained the most significant definitive treatment independently of the time of surgery for overall and recurrence-free survivals (Table 5). To further assess these outcomes independently of OLT treatment, we omitted OLT-treated patients (n = 99) in all 3 groups. At a median follow up of 13 months, we found that NAFLD-HCC patients had a higher overall survival compared to HCV (adj. HR = 0.40, 95% CI 0.17–0.98, p = 0.0440) and a trend for improved overall survival compared to the HBV group (adj. HR = 0.40, 95% CI 0.16–1.06, p = 0.0664), consistent with the previous models. Although, there was also a trend towards higher recurrence-free survivals in the NAFLD-HCC patients compared to HBV and HCV, these were no longer significant (Supplemental Table 1, Supplemental Fig. 1).

## Discussion

We present the largest detailed NAFLD-associated HCC cohort with long follow-up to date. Important clinical differences between NAFLD and viral etiologies of HCC were identified, including that HBV-associated HCC patients present at a younger age and have larger tumors at the time of presentation, which lends them to be outside of OLT criteria. Although NAFLD patients tend to have more decompensated liver disease at the time of HCC presentation, the overall survival rates are higher when compared to HBV and HCV, independently of OLT as the most definitive treatment. NAFLD-HCC patients had a higher trend towards recurrence-free survival rates compared to HBV and HCV HCC patients.

Our report is the first one conducted in a Region 5 of the transplant allocation geography where patients tend to have higher Model For End-Stage Liver Diseases (MELD) at the time of transplantation^32^. These geographical variabilities create important population differences and therefore outcomes when comparing studies. Hester *et al*. recently analyzed the outcomes of a group of 97 NASH HCC patients. In their study, when compared to HBV, HCV and alcoholic-associated liver (ALD) disease, NASH HCC patients had worse overall survivals compared to ALD patients but similar survival rates as HCV or HBV cases (median follow up time of 16 months)^14^. Wakai *et al*. evaluated post-surgical outcomes in 17 NAFLD-associated HCC cases and demonstrated that although the overall survival was not different between NAFLD, HBV and HCV patients, the recurrence-free survival was improved in the NAFLD cohort at a median follow-up time of 87 months^15,33^. We found similar trends in our cohort, although our data may have been limited by a smaller sample size with a shorter follow up period after adjusting for OLT patients. Other than geographical differences, sample sizes, and length of follow-up times can explain the differences in our findings. Our results and others’ also further validate the heterogeneity of NAFLD-HCC cases in biology and ascertainment of cases in studies given the lack of biomarkers for NAFLD and NASH diagnoses.

HCC in the non-cirrhosis liver has been reported to occur in NAFLD^10,12^. Since distinguishing NAFLD, NASH and different stages of fibrosis remains a diagnosis based on pathology, assessing liver histology in NAFLD-associated cases of HCC is critical but is often lacking in larger studies. Our detailed pathological analysis enabled us to distinguish between cirrhosis/advanced fibrosis cases when compared to non-cirrhosis. We report that ~18% of our cohort did not have any cirrhosis, although our definition was conservative due to including all bridging fibrosis cases (F3 and F3-F4 by METAVIR) in the advanced fibrosis/cirrhosis group. We found that patients who self-identified as Hispanic and had a larger BMI are more likely to develop HCC in a cirrhosis background. We also noted that the cirrhosis HCC group was less likely to have dyslipidemia or be on statin therapy (16% in the cirrhosis group versus 25% in the non-cirrhosis group) although this was no longer significant after adding BMI in our model. We interpret these data several ways. One possibility is that patients with advanced fibrosis/cirrhosis have a different lipoprotein metabolism and therefore lipid profile compared to patients without advanced liver disease^34,35^. Another potential reason is that the observed effects are related to statins since their use has been shown to decrease fibrosis progression and HCC^12,36^, although the small sample size of statin users precluded further sub-group analyses to confirm this. Few studies have attempted to differentiate statin effects between those with and without cirrhosis. In a recent case-control study comparing cirrhosis and non-cirrhosis cases based on histology, dyslipidemia (as defined by a high LDL cholesterol or triglycerides) was independently associated with HCC development in the non-cirrhosis group (adjusted OR = 1.74, p < 0.05)^37^. Although the cirrhosis group had a larger BMI (29.2 kg/m^2^) when compared to the non-cirrhosis group (26.1 kg/m^2^), those differences were not significant (p = 0.05), which is possibly explained by only having 28 NAFLD patients in the cohort of 545 individuals (5%). Statin use has also been shown to be associated with a decreased HCC mortality, although again in most studies NAFLD cases only comprise a small group of the patient population^38,39^. Teasing out the effects of dyslipidemia and statin use in different ethnic backgrounds will be important for future studies with larger samples sizes given our findings.

### Limitations

While our study highlights important differences between outcomes of NAFLD HCC (cirrhosis and non-cirrhosis) compared to HCV- and HBV-associated HCC, there are limitations. UCLA is one of the largest tertiary-care liver transplantation center in the U.S.; therefore, the majority of our patients were referred from outside institutions, thus creating a bias towards OLT evaluation and treatment. This may explain the large proportion of decompensated NAFLD patients who received OLT (versus regional differences in transplantation allocation where Region 5, which includes California, tends to have a sicker patient population^40^). However, this also allowed for a more diverse patient population due to the large referral pattern seen in Los Angeles. Being in a transplant center also provided for a detailed review of the pathology, which is often lacking in large cohorts. To the best of our knowledge, there are no previous free language processing approaches that would have identified cases within the EMR. This approach also allowed us to minimize selection bias of only studying patients seen by hepatologists and therefore have OLT or other curative treatments offered. Inter-observer differences between radiographic assessments of HCC also introduced differences in tumor measurements given that most initial imaging studies were done outside of UCLA. We attempted to normalize these by only including CT or MRI studies that were re-interpreted at UCLA using the validated Li-RADS score. Another limitation is the small sample size of the non-cirrhosis cases of HCC (23), restricting further analyses, such as tumor differences, and teasing out the effects of dyslipidemia and statin treatment. This is especially relevant because recent obese mouse models have demonstrated that NASH HCC can occur through independent mechanisms of NASH^41^.

## Conclusions

In conclusion, we present a large, diverse NAFLD HCC patient population with detailed clinical and pathological data, not only allowing for important differences to be identified between various stages of fibrosis but also comparing these to viral etiologies of HCC. Identifying population-specific biomarkers, which will likely require a combination of clinical risk factors, laboratory data and tumor growth data, will be important in this group of patients. These studies also support the use of longitudinal biomarker studies to identify potentially useful diagnostic targets. The association between BMI and dyslipidemia remains of crucial clinical significance due to the non-cholesterol and pleiotropic effects of statins on HCC and liver fibrosis. This provides an avenue for statin use as a chemoprotective agent not only in NAFLD cirrhosis patients but specifically in the sub-group of non-cirrhosis patients who are not currently being targeted for screening. New prospective studies are needed to assess the benefits of statins in the non-cirrhosis group, which comprises a large portion of the NAFLD.

## Supplementary information

Supplemental information.
