# Supplementary material for: Improved survival after treatments of patients with nonalcoholic fatty liver disease associated hepatocellular carcinoma
Source: Sci Rep. 2020 Jun 18;10:9902. doi: 10.1038/s41598-020-66507-7 (PMC7303220; doi:10.1038/s41598-020-66507-7)

Improved survival after treatments of patients with nonalcoholic fatty liver disease associated hepatocellular carcinoma

Jihane N Benhammou[1*], Elizabeth E Aby[2], Gayaneh Shirvanian[2], Kohlett Manansala[1], Shehnaz K Hussain[3,4] and Myron J Tong[1,5]

[1]Pfleger Liver Institute, University of California Los Angeles, Los Angeles, CA
[2]Vatche and Tamar Manoukian Division of Digestive Diseases, David Geffen School of Medicine at UCLA, Los Angeles, California, USA.
[3]Department of Epidemiology, Fielding School of Public Health, University of California, CA
[4]Department of Medicine, Samuel Oschin Comprehensive Cancer Institute, Cedars-Sinai Medical Center, Los Angeles, CA
[5]Liver Center, Huntington Medical Research Institutes, Pasadena, CA

Elizabeth E Aby
Abyxx002@umn.edu
Division of Gastroenterology Hepatology and Nutrition
420 Delaware Street SE
MMC 36
Minneapolis, MN 55455

Gayaneh Shirvanian
gayanehshirvanian@yahoo.com
16830 Kingsbury Street, Granada Hills, CA 91344

Kohlett Manansala
kohlettm88@gmail.com
20118 Livorno Way Porter Ranch, CA 91326

Shehnaz K Hussain
Shehnaz.hussain@cshs.org
Cedars-Sinai Medical Center
700 N. San Vicente Blvd Pacific Design Center G599, West Hollywood, CA 90069

Myron J Tong
mjtsp@aol.com
Pfleger Liver Institute
200 UCLA Medical Plaza, Suite 214, Los Angeles, CA 90095

*Corresponding author: jbenhammou@mednet.ucla.edu
Jihane N Benhammou
Pfleger Liver Institute
200 UCLA Medical Plaza, Suite 214, Los Angeles, CA 90095

<u>Appendix</u>:

<u>JCCC and UCLA EMR</u>

1. Males and females
2. Age ≥18 years at the initial diagnosis of HCC and NAFLD and/or NASH
3. Dates 01/01/2000-12/31/2016
4. Diagnosed with Hepatocellular Carcinoma (HCC)
   a. Either listed on JCCC Cancer Registry list (~3k patients)
   b. <u>OR</u> has diagnosis of Hepatocellular carcinoma (HCC) by EMR as defined by:
      1. ICD-9 or ICD-10: (155.2, 155.0) [C22.8, C22.0, C22.7]

AND DIAGNOSED with one of the following comorbidities:

5. Non-alcoholic fatty liver disease (NAFLD) patients
   a. NAFLD by (ICD-9) or [ICD-10]: (571.8, 571.9, 573.8, 573.9) [K76.9, K76.0, K76.89, K76.81]
6. <u>OR</u> Non-alcoholic steatohepatitis (NASH) patients
   a. NASH by (ICD-9) and [ICD-10]: (571.8, 571.9, 573.8, 573.9) [K75.81, K75.8, K75.9]
   b. NASH by pathology natural language processing using the following keywords: (NAFLD Activity Score (NAS) and include fibrosis score F0-F4):
   c. "steatohepatitis", "ballooning", "NAFLD activity score", "NASH" or "NAFLD"
7. <u>OR</u> metabolic syndrome (Please flag metabolic syndrome patients)
   a. Metabolic syndrome defined by ICD-9 or ICD-10: (277.7) [E88.8]
   b. OR Metabolic syndrome as defined by: diabetes [(ICD-9: 250.00) [ICD-10: E11]: Diabetic nephropathy N08.3, Diabetic polyneuropathy G63.2, Diabetic retinopathy H36.0], hypertension [portal hypertension (572.3 or K76.6) or Hypertension (401.9)] and hyperlipidemia (272.4) or [E78.4, E78.5]

ADDITIONALLY,
Evaluated all EMR patient from oncology liver clinic and all patients who had hepatic resection from 2010-2016 (separate lists).
Abstracted medications, A1c, liver tests, AFP level, all radiology data, all pathology data.

All EMR data was ranked based on following keywords: "steatohepatitis", "ballooning", "NAFLD activity score", "NASH", "NAFLD" or "metabolic syndrome"

Supplemental data:

Data source

EMR cases were identified using *International Classification of Diseases* (ICD-9 and ICD-10 codes) for HCC with a diagnosis of NAFLD, NASH, the metabolic syndrome, or features of the metabolic syndrome as defined by diabetes (including its complications diabetic nephropathy, diabetic polyneuropathy, diabetic retinopathy), hypertension, and dyslipidemia (see appendix). Given the under-reported cases of NAFLD and NASH using EMR ICDs [41,42], we identified additional cases using natural language processing of all pathology, operative, diagnostic and interventional radiology (using *Current Procedural Terminology*, CPT codes) reports with the following key terms: "NASH", "NAFLD", "steatohepatitis", "ballooning" or "NAFLD activity score". HBV and HCV cases were used as a comparative group and were identified within the Los Angeles area from the Liver Cancer Center in Pasadena, CA. HBV and HCV patients received their care at the local clinic and/or at a tertiary transplant center including at UCLA Medical Center [19].

Detailed NAFLD-, HBV-, and HCV-HCC case definitions

3,358 HCC cases were identified in the JCCC cancer registry and 4,809 cases were identified from the EMR abstraction using ICD codes and natural language processing. To select only the validated cases identified through these databases, key words from natural language processing (steatohepatitis, ballooning, NASH, NAFLD, and metabolic syndrome in that order) were counted by the number of times they appeared in a suspected case's chart. Individuals were then ranked based on those numbers, and individual patient chart reviews were conducted to validate these definitions. All highly ranked "NASH" patients were evaluated, followed by all highly ranked "NAFLD", "steatohepatitis", "ballooning", "NAFLD activity score" and "metabolic syndrome"

cases. We defined highly ranked individuals as anyone who had the word “steatohepatitis” mentioned at least twice in their chart with any one of the other terms listed above. To assess the possibility of false negatives, a total of 10-15 charts were reviewed in patients who had a key term appear once or twice (without other key terms), 10-15 cases were reviewed in patients who only had one key term appear, and an additional 20 charts were reviewed without any key terms; no cases were identified through these chart reviews. Using the above algorithm, 430 charts were reviewed. Of those suspected cases with high ranking, 127 met inclusion criteria (see below). Two patients were removed in the final analysis: one patient was removed due to being the only one having received stereotactic body radiation therapy; another patient was removed due to an unclear sequence of events related to the diagnosis of HCC (outside scans only available for review) and only a small focus on explant that was not consistent with the imaging data (<0.5 cm without field defect from prior therapies).

Supplemental table 1- Cox multivariable analysis of patients and treatment variables associated with overall survival and recurrence free survival after omitting all OLT patients (n=355).

Variable	HR	95% CI	*P* value
Overall Survival			
Male gender	1.14	0.85-1.54	0.3882
Age (per year)	0.98	0.97-1.00	0.0069
Etiologies:			
HCV vs HBV	1.02	0.70-1.46	0.9307
NAFLD vs HBV	0.41	0.16-1.01	0.0664
NAFLD vs HCV	0.40	0.17-0.98	0.0440
Race/Ethnicity			
African American	Ref	-	-
White	0.78	0.23-2.62	0.6872
Asian	0.77	0.24-2.51	0.6664
Hispanic	0.94	0.27-3.26	0.9175
Not Hispanic	1.08	0.21-5.56	0.9302
Most definitive treatment:			
Chemotherapy	Ref	-	-
PEI	0.36	0.14-0.92	0.0338
Resection	0.15	0.08-0.29	<0.0001
RFA	0.16	0.08-0.30	<0.0001
TACE	0.45	0.26-0.79	0.0055
Supportive care	0.83	0.49-1.42	0.5000
Recurrence Free Survival			
Male gender	1.12	0.90-1.51	0.245
Age (per year)	0.99	0.98-1.01	0.0626
Etiologies:			
HCV vs HBV	1.08	0.78-1.49	0.6504
NAFLD vs HBV	0.69	0.39-1.39	0.3002
NAFLD vs HCV	0.64	0.34-1.20	0.1630
Ethnicity:			
African American	Ref	-	-
White	0.76	0.27-2.18	0.6104
Asian	0.91	0.33-2.53	08608
Hispanic	0.92	0.31-2.71	0.8734
Not Hispanic	0.84	0.24-2.93	0.7824
Most definitive treatment:			
Chemotherapy	Ref	-	-
PEI	0.35	0.13-0.91	0.0313
Resection	0.26	0.13-0.41	<0.0001
RFA	0.27	0.15-0.48	<0.0001
TACE	0.52	0.30-0.90	0.0200
Supportive Care	0.72	0.42-1.22	0.2173

Harrell's C-statistic= 0.782 for the overall survival and 0.721 for the recurrence-free survival.

Supplemental figure 1-Overall and recurrence free survival of NAFLD, HBV and HCV cases without OLT as definitive treatment. A. Overall survival of the three groups; B. Recurrence free survival for all three groups. The figure is adapted from Benhammou et al. From Bedside to Bench-side: the Clinical, Epidemiological and Molecular Basis for Nonalcoholic Steatohepatitis and Hepatocellular Carcinoma. *UCLA*[24].

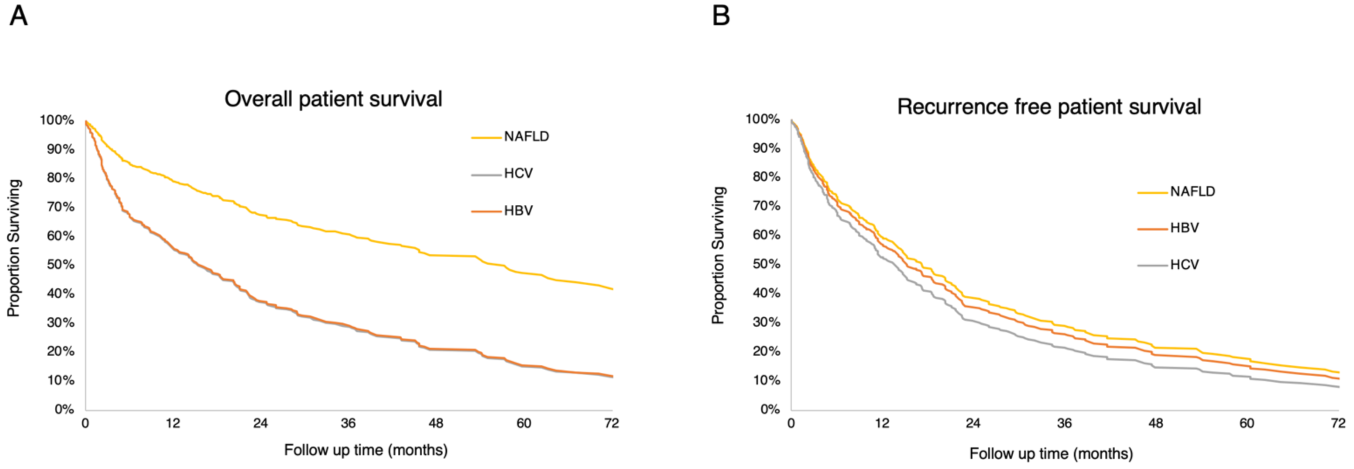

Supplement: Supplementary file 1 — Supplemental information. [file 41598_2020_66507_MOESM1_ESM.pdf]
